# Review on Metal Powder Manufactured by Technologies Utilizing Centrifugal Force

**DOI:** 10.3390/ma18214905

**Published:** 2025-10-27

**Authors:** Zhining Wu, Xianke Lu, Qi Shi, Yuyuan Zhao

**Affiliations:** 1 School of Mechanical and Automotive Engineering, Ningbo University of Technology, Ningbo 315211, Chinashiqi@nbut.edu.cn (Q.S.); 2 Yizumi Holdings Co., Ltd., Guangdong Provincial Key Laboratory of Intelligent Molding Equipment Enterprises (YIZUMI), Foshan 528300, China

**Keywords:** metal atomization, plasma rotating electrode process, centrifugal atomization, disintegration mode, powder characterization

## Abstract

High-quality powders with spherical particles and controllable properties can be produced using centrifugal force. This review provides a comparative analysis of two centrifugal atomization techniques: the plasma rotating electrode process (PREP) and centrifugal atomization (CA). It systematically examines the fundamental principles, film disintegration modes, and the resultant powder characteristics, with a focus on mechanisms that lead to common defects. By evaluating current technological limitations and highlighting potential pathways for advancement, this review aims to offer valuable insights for the future development of high-quality, spherical metal powders for advanced manufacturing applications.

## 1. Introduction

With the rapid development of additive manufacturing (AM) and powder metallurgy (PM), the mechanical [1,2], thermoelectric [3], and physical properties [4] of metal and alloy parts can be effectively tuned and enhanced. High-quality metal powder, particularly with a spherical shape, as the raw material, is becoming more and more important for consistent processability and superior final part performance [5,6]. Atomization is deemed the most appropriate approach to manufacture powders suitable for AM industries [7,8,9]. Contamination-free and accurate control of particle size distribution makes atomization the most popular way for the commercialization of spherical metal powder manufacturing.

**Figure 1:**
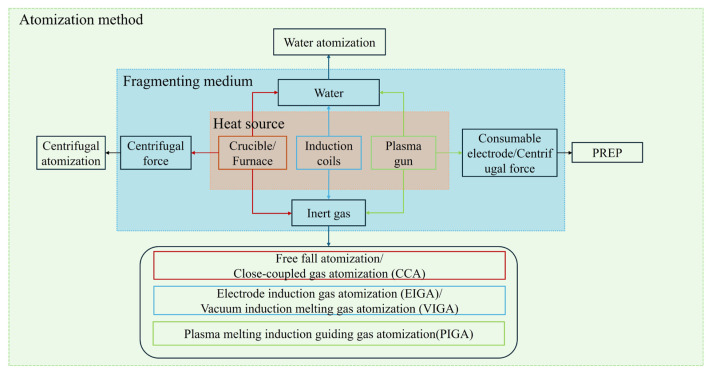
Schematic illustration of the atomization processes.

The atomization techniques can be classified into two groups. The main difference between them is whether the formation of particles is derived from external media or not. The external medium-based atomization processes typically employ gas or water. Conventional gas and water atomization techniques are widely employed for their high throughput and cost-effectiveness, but they often introduce inherent limitations. These methods, which rely on the disintegration of a molten stream by high-velocity fluids, can lead to the formation of irregular, teardrop-shaped particles, internal porosity, and satellite particles, subsequently compromising powder performance [10,11]. It is precisely to address these shortcomings that centrifugal force-based powder manufacturing technologies have emerged as a critical advancement. Techniques such as the plasma rotating electrode process (PREP) and centrifugal atomization (CA) are fundamentally necessary as they utilize centrifugal force to eject molten metal from the periphery of a rapidly rotating electrode or disk. This mechanism of droplet formation, governed by surface tension and rotational kinematics, inherently produces highly spherical, dense, and contamination-free powders with a narrow size distribution and minimal internal defects. The general atomization process, including heat sources for feedstock melting and fragmenting media, is depicted in Figure 1.

This article gives a thorough review of the PREP and CA processes. Firstly, a detailed description of each method, along with important processing parameters, is provided. Secondly, the disintegration modes and the models used for the prediction of particle size distribution are classified and analyzed. Lastly, the benefits and drawbacks of PREP and CA are discussed.

## 2. Atomization Processes

### 2.1. Plasma Rotating Electrode Process (PREP)

**Figure 2:**
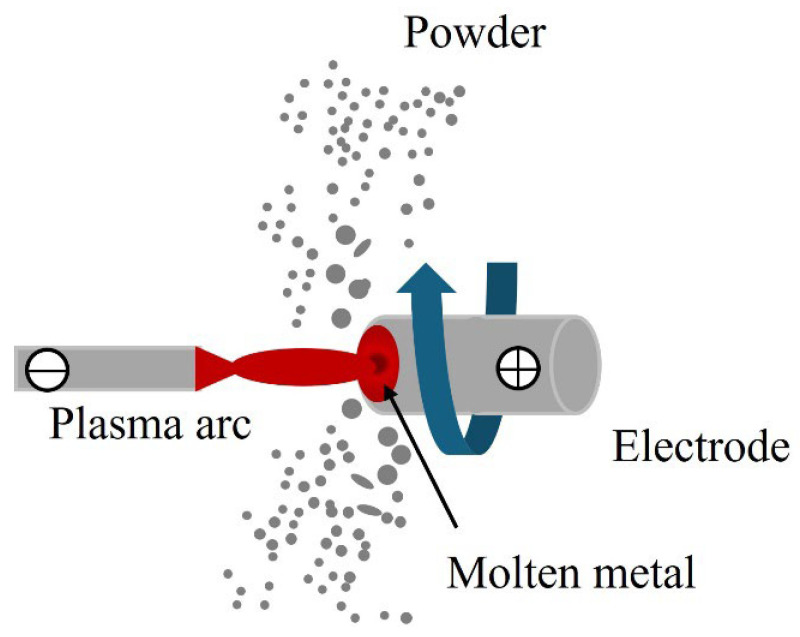
Schematic of the REP.

In PREP, the feedstock must be prepared into a rod with a specific diameter. The rod is fixed and rotates at a high speed and acts as an electrode, as shown in Figure 2. A plasma arc is produced from the cathode to melt the rod’s end. The molten metal is then sheared and spun off by the centrifugal force to create droplets. Surface tension allows for the generation of spherical particles during the droplet cooling process. The quality of the as-manufactured powder is evaluated according to the degree of sphericity and particle size distribution, which are critical for a variety of PM and AM applications.

The flowability of powder is an essential requirement for use in AM. Poor flowability is detrimental to the quality of the product, resulting in defects due to uneven spreading of powder on each layer or even failure during printing. The most effective way to improve the flowability of the powder is to increase particle sphericity, which is influenced by many factors. Li et al. [12] produced Al_0.5_CoCrFeNi powder using PREP, intended for AM use. A considerable amount of irregularly shaped particles was produced, which was mainly attributed to surface tension turning molten droplets into flakes, and the collision between solidified particles with overlapping trajectories. The sphericity was improved by adjusting the rotational speed and optimizing the cooling efficiency of the molten metal via an aiding inert gas. Han et al. [13] examined the Ni-11Mo-8Al-3Ta-2Cr-1Re powder produced by PREP and found that large particles (>150 μm) were irregularly shaped and contained more content of Al, N and O. Possible reasons for the reduced sphericity are the existence of microsegregation zones in the raw materials and the incomplete melting in the Al-rich regions during atomization. The same trend was noticed in the Nitinol powder manufactured by PREP [14], which tended to have a flat-shaped morphology for particles over 500 μm. This may be explained by the insufficient flight distance before the molten droplets strike the chamber wall. The formation of irregular powder in PREP typically results from a combination of material properties, uneven droplet temperature, and insufficient solidification flight paths. Table 1 illustrates critical size and types for the formation of irregular particles.

Fine particles are subject to stronger molecular and electrostatic forces, reducing their flowability. Furthermore, their high specific surface area encourages adsorption, agglomeration, and cohesion, resulting in a higher repose angle [15]. Moreover, tap and apparent densities serve as key indicators, providing insight into the sphericity, particle size distribution, and the coefficient of friction between particles. Generally, the greater the ratio of the tap density to apparent density, the better the flowability of the powder. The relevant properties of the different powder types are summarized in Table 2.

**Table 1:** Critical size and causes for the formation of irregular particles.

Type	Critical Size for Irregular Particles (μm)	Shape	Causes of Irregular Particles	Ref.
Ni-11Mo-8Al-3Ta-2Cr-1Re	>150	wrinkled	Only fully melted droplets can spheroidize into spheres, driven by surface tension. Incomplete melting inevitably leads to irregular particle shapes.	[13]
TiAl	>150	non-spherical	The low electrode rotation speed during the initial stage generated insufficient centrifugal force on the liquid films.	[16]
Ti6Al4V	>250	flat	Semi-solid liquid films are torn away and solidify into irregular shapes before surface tension can spheroidize them.	[17]
316 steels	>150	-	Surface peeling from semi-solid films generates irregular particles; the extent of peeling and mass fraction of such powder depend on specific properties of different alloys	[15,18]
CoCrMo	>150			
Ni_3_Al-based IC21		jagged	Centrifugal force ejected core defects with high melting point which resisted spheroidization, forming irregular particles that retained their initial morphology.	[19]

Particle size distribution is another key factor in determining the merits of powder in applications. One of the research and development priorities of PREP, therefore, is the precise control of particle size distribution. Cui et al. [29] found that the mode of the particle size distribution of Ti64 decreases with increasing rotational speed or increasing electrode diameter, cooling rate has a significant influence on particle sizes, and droplets can agglomerate during flight. Another study [30] further examined the impacts of gas type and flow rate on particle sizes. They found that the particle sizes were influenced by the gas used, and they initially decreased and then slightly increased as the gas flow rate increased. These phenomena are caused by two effects of the gas on the creation and solidification of the liquid droplets, namely the disturbance effect and the cooling effect. The disturbance effect refers to the mechanical impact between the gas and the molten metal, while the cooling effect refers to the convective heat transfer between the gas flow and the melt droplets. Finer powder was manufactured using He compared with using Ar, because of the higher thermal conductivity of He (0.1442 W/m·K) than Ar (0.0164 W/m·K) and therefore more rapid cooling. Zhao et al. [31] investigated the changes of particle size of both Ti64 and SUS316 powders under varying PREP parameters. The particle size increased with increasing gas flow rate and increasing arc current. The influence of gas flow rate is manifested in the disturbance and cooling effects. The gas blasts accelerate the cooling of the molten metal and cause a disturbance to the formation of melt ligaments. The change in temperature alters the thermophysical characteristics of the melt, which impede the granulation process and cause coarser particles. The arc current represents the output power of the arc and directly determines the melting rate. Varying melt rate changes the thickness of the melt film on the electrode and the melt disintegration mode, resulting in varying particle sizes. In addition to process parameters, the physical characteristics of the raw materials are also critical in determining particle size. Under the same operating conditions, the particle size of the Ti64 powder was always greater than that of the SUS316 powder, because the differences in density, viscosity, and surface tension result in distinct centrifugal granulation behaviors during PREP. In laser powder bed fusion (LPBF) process, fine powder, where van der Waals forces dominate interparticle interactions, exhibits increased agglomeration and reduced flowability. This results in the formation of numerous uneven voids and gaps during powder spreading, significantly decreasing powder bed density. In contrast, coarse powder, with large geometric interparticle gaps that cannot be fully filled, achieves a slightly lower packing density than medium-sized powder after stabilization [32]. Powder with excessively large or small particle sizes, or an overly uniform particle size distribution, will experience reduced flowability, which in turn can reduce powder bed density and generate pore defects in LPBF samples [6]. The particle sizes of various metal powders produced by PREP under a range of processing parameters are summarized in Table 3.

**Figure 3:**
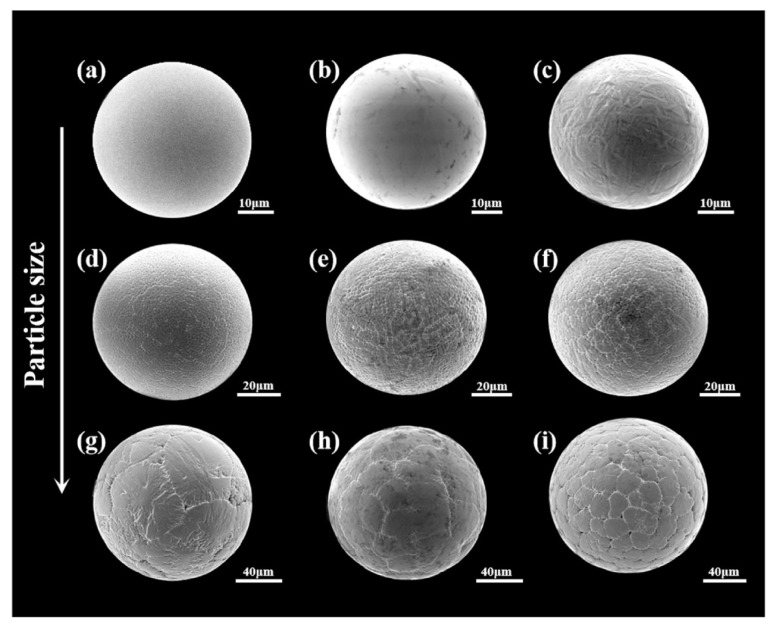
Surface morphologies of TiAl powder with particle sizes of (**a**–**c**) 50 ± 10 μm, (**d**–**f**) 80 ± 10 μm, (**g**–**i**) 130 ± 15 μm [37].

Altering the cooling rate, through adjusting various processing parameters, can generate different surface morphologies and microstructures with changing particle size. Yang et al. [37] observed that a high cooling rate in PREP can cause a smooth surface and inhibit element segregation of TiAl alloys, as shown in Figure 3. The microstructure changed from smooth to dendritic and equiaxed morphologies as the particle size increased. Chen et al. [38] conducted a similar investigation into the effect of cooling rate on phase transformation in TiAl powder. Eutectoid decomposition of B2-NiTi occurred in particles larger than 178 μm due to a slowed cooling process, and consequently, a greater number of secondary phases was detected.

### 2.2. Centrifugal Atomization (CA)

**Figure 4:**
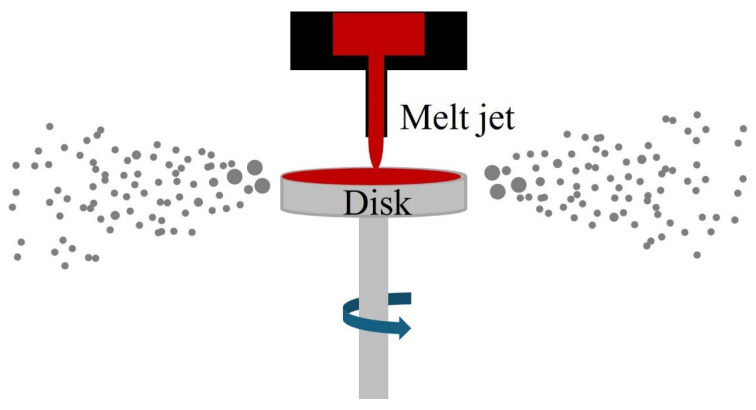
Schematic of the CA process.

CA, originally developed from the spray forming process, is an efficient way to manufacture metal or alloy powders with spherical morphology and narrow particle size distribution. It utilizes centrifugal force, rather than external media, to atomize the melt jet. Consequently, gas-entrapment porosity in the particles is eliminated, and the manufacturing cost is reduced. The primary distinction between CA and PREP is, as illustrated in Figure 4, that a high-speed spinning disk or cup is employed in CA to break up the impinging melt jet.

The force that breaks up the molten metal during the atomization process is the centrifugal force, which can be expressed by the following equation:

(1) $$F=m\cdot r\cdot \omega^{2}$$

where m is the mass, r is the radius, and ω is the angular velocity of the element of the melt in consideration. The geometry and rotating speed of the rotating disk, as well as the physical properties of the molten metal, are therefore the most vital parameters to determine the quality of the as-produced powder. The study by Suastiyanti et al. [39] showed that a higher rotation speed resulted in a higher yield of powder production, due to less accumulation of molten Sn on the disk. Increasing the rotating speed generated Sn powder with a smaller particle size, due to the increased centrifugal force imparted by the disk to the molten Sn. A similar pattern of particle size distribution was found in another study by Li et al. [40] for the aluminum powder. They also showed that a transition from a unimodal to a bimodal particle size distribution occurs when the rotating speed exceeded 33,000 rpm, because the main liquid droplets are coterminous with a necked-down column of liquid metal, which then separates into smaller droplets when the major spheroids are freed. Using a hybrid atomization system, Salges et al. [41] demonstrated that increasing the peripheral gas flow rate reduced the mean particle size of copper powder. This effect was most pronounced at low rotational speeds and was linked to changes in disk-particle dynamics. The contribution of the external gas flow becomes prominent when the velocity of the molten copper is slow at the edge of the disk with a low rotation speed. The particle size of various metal powders produced by CA under a range of processing parameters are summarized in Table 4.

Generally, CA-produced powder is less spherical than PREP powder, primarily due to three factors. First, fine droplets may cool too quickly to fully spheroidize before solidifying, while extended liquid films can delay spheroidization. Second, oxidation in the chamber can form rigid oxide layers on droplets, hindering spheroidization. Using an inert atmosphere minimizes this issue. Third, secondary atomization upon wall impact can distort larger droplets. Smaller droplets solidify quickly and remain spherical, while larger ones may splatter into flakes. Tian et al. [45]. found that the critical particle size for calcium to stay spherical during CA process is 177 μm. Larger droplets form irregular shapes due to incomplete spheroidization before solidification or distortion from impacting the quench bath while still semi-solid. Denmud et al. [49]. investigated the relationship between the particle’s morphologies and oxygen content. An oxygen content exceeding 0.05 vol.% led to the appearance of irregular powder due to increased surface oxidation.

The cooling rate of the atomized droplets play an important role in determining the ultimate quality and microstructure of the powder product. Droplet cooling is mainly affected by the forced convection and radiation during flight [50]. Moreover, the microstructure and phase ratio of the particles are influenced by the cooling process of the droplet [51]. A refined dendritic structure with finer secondary dendrite arm spacing (SDAS) was found in the Al-Cu alloy powder with a higher cooling rate, achieved with an Ar atmosphere instead of He, because of their different thermophysical properties [43]. Similarly, a higher cooling rate was found to suppress the formation of α-Fe in the Nd-Fe-B alloy powder, leading to an increased coercivity of the resultant magnet [44]. It was observed that the melt on the atomizer experienced faster cooling towards the edge and slower cooling towards the center, whereas a lower cooling rate appeared at the trailing edge of the droplets [52]. The temperature differences of the melting on the atomizer can yield particles with irregular shapes. In order to eliminate the influence of uneven temperature distribution, a laser was found to be efficient as an aided source of energy to melt the 316L grade stainless steel by overcoming cooling induced by convection at the edge of the melt [53]. Guo et al. [54] used Newton’s cooling model to demonstrate that reducing the cooling rate during solidification yielded smaller and more spherical aluminum particles. SDAS is a critical microstructural parameter that fundamentally governs the mechanical properties of as-solidified materials [55]. The refinement of SDAS generally leads to the improvement of the strength, toughness, and hardness of materials [56]. Understanding the SDAS-cooling rate correlation is critical for controlling solidification microstructure and subsequently improving parts properties. Research shows that a higher powder cooling rate results in a finer SDAS [56,57]. The cooling rate in PREP and CA processes is governed by factors such as cooling gas, material properties, and droplet size. The resulting microstructure varies with particle size due to differences in the droplet’s flight distance and condition before solidification. Li et al. [12] found the microstructural evolution with decreasing particle size of the Al_0.5_CoCrFeNi powder. The powder with size of 93.3 μm initially forms dendrites. At 78.6 μm, increased supercooling refines the structure into smaller dendrites/columnar crystals. Finally, at 16.7 μm, the high cooling rate from a larger gas-contact area produces equiaxed crystals. Yin et al. [24] showed that smaller particles exhibit higher cooling rates due to more rapid heat extraction than their larger counterparts during atomization. This thermal gradient also exists within individual particles, leading to a finer dendritic microstructure in the outer shell and coarser equiaxed grains in the core. Furthermore, in sufficiently fine particles, dendritic growth is strongly suppressed, resulting in a uniformly refined microstructure. The relationship between particles size, SDAS and cooling rate are listed in Table 5.

## 3. Disintegration Modes and Prediction of Particle Size

The required particle size of the powder depends on the application and varies significantly. For example, the general size distribution for laser powder bed fusion is 15 to 53 μm [61]; the most commonly used particle size in electron beam melting is 45 to 105 μm [62]; the usable particle size distribution for traditional powder metallurgy (field assisted sintering technology, hot isostatic pressing, etc.) is quite broad, ranging from 10 to 200 μm [63]; the optimal particle size for metal injection molding is 2–20 μm [64]. Therefore, if the producible particle size ranges of PREP and CA powders can be predicted based on the used raw materials and production parameters, the efficiency and practical values of PREP and CA will be significantly enhanced.

The underlying disintegration mechanisms in centrifugal atomization processes need to be considered to understand the correlations between particle size and operation conditions. The formation of particles is affected by two actions during the centrifugal granulation process: cooling and disintegration of the liquid. The effect of cooling rate on particle size has been discussed in the previous section. A higher cooling rate increases the viscosity and surface tension of the molten metal, and a larger viscous force is required to break up the melt, which results in particles with a larger size [31].

**Figure 5:**
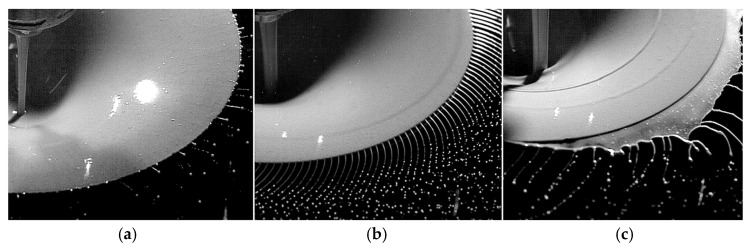
Three liquid disintegration modes on a flat disk with different volume flow rates and rotational speeds: (**a**) direct drop mode; (**b**) ligament mode; (**c**) sheet mode [65].

Figure 5 illustrates the three disintegration modes in the centrifugal granulation process: sheet (or film), ligament, and direct drop formation, which are applicable regardless of the type of liquid.

In the sheet disintegration mode, the molten metal forms a thin film covering the whole surface of the rotating disk. The film gradually disintegrates into ligaments or droplets as it is flung outward over increasing distance. In the ligament mode, the molten metal fragments are created by centrifugal and shear forces at the edge of the rotating disk. The ligaments gradually turn into droplets after detaching from the disk and traveling a certain distance. In the direct drop formation mode, droplets form directly at the edge of the rotating disk, due to more effective shear separation, and immediately solidify into solid particles.

The transition from direct drop mode to ligament mode, and from ligament mode to sheet mode, occurs when the wave pattern of the film flowing on the surface of the rotating atomizer changes [66]. The instability of the film is developed along the radial direction of rotation, resulting in the disintegration into rivulets and drops. With the influence of centrifugal force, the dominating mechanism to form the wave covering a spinning disk is the Rayleigh-Taylor instability, which is also the cause for ligament disintegration at the disk rim [67,68]. The wave patterns become complicated with the increase of flow rate and rotation speed. Four different wave zones can be distinguished along the radius from the center to the rim [69,70]: (1) concentric waves at the center of the disk; (2) first laminar wave; (3) turbulent regime defined by disordered ripples on the free surface; (4) second laminar wave with fine scale surface perturbations. Irregular wave patterns are also observed with various combinations of flow rate and rotation speed [71]. The discrepancy in different research can be attributed to the different and complex combinations of operation parameters, including disk geometry, flow rate, rotation speed, and physical properties of the liquid.

Dimensionless numbers in fluid mechanics are usually taken into consideration to investigate the comprehensive effects of the operation conditions, as well as to account for the influences of inertial, viscous, and capillary forces on the wave patterns in the thin film on a spinning atomizer. Reynolds number (*Re*), Weber number (*We*) are two of the most widely used dimensionless numbers in fluid mechanics. *Re* predicts whether a flow is laminar or turbulent, while *We* predicts the stability of the interfaces between different fluids. Peng et al. [72] found that the transition from direct drop mode to ligament mode and from ligament mode to sheet mode occurred when the liquid flow rate, rotation speed, density, and viscosity of the liquid were increased. The transition criteria between different disintegration modes are associated with a nondimensional critical volume flow rate $Q^{*}$, which is related to *We* and *Re* as follows:

(2) $$Q^{*}=K^{*}{We}^{m}{Re}^{n}$$

where $K^{*}$, *m* and n are constants. $We=\rho \omega^{2}D^{3}/8\sigma$ and Re=ρQ/μD, by the definition. Therefore, the transitions are dependent on fluid density (*ρ*), fluid viscosity (*μ*), surface tension (*σ*), flow rate (*Q*), disk angular speed (*ω*) and disk diameter (*D*).

The particle size of the powder manufactured by the rotary cup was also found to be related to the dimensionless numbers *We* and *Re* [73]. Moreover, the Ohnesorge number (*Oh*), which is used to describe the tendency for a drop to coalesce or fragment, was introduced to measure the stability of liquid blobs:

(3) $$Oh=\frac{{We}^{1/2}}{Re}=\frac{\mu }{{\left(\rho \sigma d_{L}\right)}^{1/2}}$$

where *We* and *Re* are defined by $We=\rho v^{2}d_{L}/\sigma$ and $Re=\rho vd_{L}/\mu$, $d_{L}$ is the diameter of the ligament, and v is the velocity of the liquid film released from the rotating cup. The wave number (number of ligaments) and optimal wavelength of the ligament in the Rayleigh regime can be predicted and calculated using Weber’s theory and Rayleigh’s theory. The disk-rim disintegration in the ligament mode is generated by the Rayleigh-Taylor instabilities developed by the action of centrifugal force on the film. The number of ligaments, determined by the wave number, is more related to the physical properties of the liquid, atomizer configuration, and rotation speed, whereas the shape of the ligaments is highly influenced by the liquid flow rate, with a higher flow rate leading to longer and wider ligaments [74]. The number of ligaments was also found to be decided by We and the stability number ($st=2\mu^{2}/\rho D\sigma$) [75,76]. The transition from the direct drop mode to the ligament mode occurs at a high *We*, and the diameter of the ligament is a function of the transition flow rate [77].

Moreover, the Hinze-Milborn number, a widely used dimensionless empirical parameter, has been proposed to predict the disintegration mode [29,31,78,79,80,81]:

(4) $$Hi=\frac{\mu^{0.17}Q\rho^{0.71}\omega^{0.6}}{\sigma^{0.88}D^{0.68}}$$

*Hi* is used not only for PREP and CA, but also for spray formation in general. For *Hi* < 0.07, the disintegration/spray is the direct drop mode; for 0.07 < *Hi* < 1.33, the ligament mode; and for *Hi* > 1.33, the sheet mode. It shows that the transition from direct drop to ligament mode and ligament to sheet mode can be triggered by increasing rotation speed and/or liquid flow rate and by decreasing the diameter of the atomizer for any given raw material.

There are a number of existing correlations for predicting the transitions of disintegration modes, which are summarized in Table 6, due to the different testing environments and atomizer configurations.

Research has shown that the ligament mode can produce smaller droplets with a narrow size range compared with the direct drop mode and sheet mode and is more suitable for powder manufacturing. As the rotational speed increases, it more readily leads to ligament formation, which in turn results in finer droplet sizes due to the shortened wavelength of the dilational wave [77]. A broader particle size distribution resulted from filament formation, which was mainly controlled by the particle cooling rate and liquid film breakup mode. The ligament mode yields a narrower particle size distribution, as it minimizes filament formation at higher feed rates and produces droplets with sizes largely unaffected by the cooling air rate [84]. Therefore, the influences of material properties and atomization conditions on the disintegration mode and the particle size of the as-produced powder have always been a key research focus. Numerous models and empirical formulas have been established to predict changes in powder particle size under various disintegration modes. To achieve a deep understanding of the impact of varying operating conditions on the particle size, however, it is necessary to comprehend the four stages of the evolution of the flow in the disintegration process [70,85]: (1) spreading of liquid film on the surface of the rotating atomizer; (2) unsteady free-boundary flow in the transition zone; (3) formation of ligaments at the rim of the atomizer due to Rayleigh-Taylor instability; (4) dynamics of surface-tension-induced breakup leading to the formation of droplets.

The liquid flow impinging on the atomizer spreads from the center to the rim with radial and azimuthal velocity components under the influence of centrifugal and inertial forces [86]. Besides investigating the effect of the operating conditions, such as melt flow rate, rotation speed and size of atomizer, another approach is to look at the thickness of the liquid film, which is vital to determine the rupture behavior at the atomizer’s edge, since a thinner liquid film tends to form smaller droplets [87]. The thickness of the liquid film at the rim of the atomizer increases with increasing Re and *Oh* and decreasing *We*. A correlation between the droplet size and liquid film thickness was developed as follows [88]:

(5) $$\frac{d}{R}=2.93\left(\frac{h}{R}\right)-0.0043$$

where d is the droplet diameter, R is the radius of the atomizer, and h is the thickness of the liquid film. The phenomenon of hydraulic jump, a discontinuity in flow in the radial direction, is often found at high rotation speed, which can result in an abrupt increase in the film thickness [89]. The position and the height of the hydraulic jump are influenced by the physical properties of the liquid and the operating conditions, and its existence makes the atomizer susceptible to skull formation, which can lead to deterioration of the service life of the atomizer [90]. Proper design of atomizers with a small size can eliminate this problem.

An interfacial slip between the liquid film and the surface of the atomizer is inevitable at high rotation speeds during the atomization process, due to the difference in critical surface tension, resulting in reduced film thinning [91]. The term used to evaluate the extent of slip between the liquid film and the atomizer surface is referred to as the slip ratio, which varies significantly with the types of atomizers. The slip ratio for flat disks is about 33.8%, while the slip ratio for cups is between 18.2% and 41.1%, depending on the cone angle [92]. Most cup atomizers have less slip than flat disk atomizers, and the transition from the sheet mode to the ligament mode occurs earlier because of the higher exit liquid velocity. However, the interaction between the cup wall and the liquid reduces the radial distance for the film to spread, resulting in a thicker film and promoting the formation of larger droplets [93]. Moreover, the slope angle increases flow resistance and amplifies unstable waves on the liquid film, which in turn generates premature rupture. From the mode transition and film thickness perspectives, therefore, disk atomizers can produce better performance than cup atomizers and yield smaller particles.

**Figure 6:**
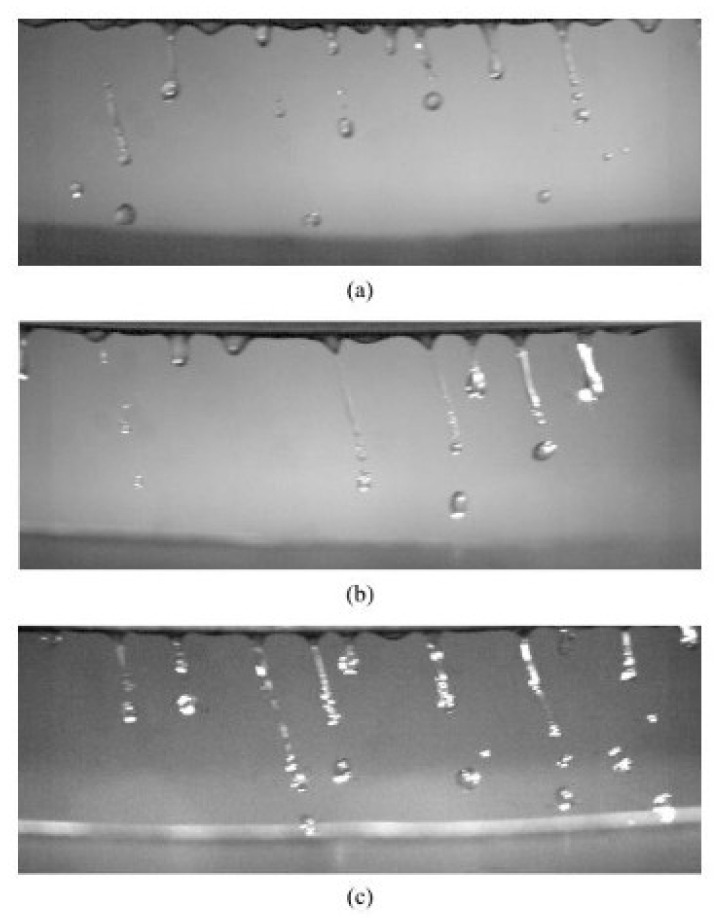
Formation of droplets for disks with edge bevel angles of (**a**) 75°, (**b**) 56°, (**c**) 36° [96].

The surface texture and edge profile of the atomizer can affect the breakup mode and thus change the droplet characteristics. Four types of flat disk, namely coarse grooved disk, fine grooved disk, disk with blocks and grooves, and disk with pits in spiral pattern, were used to investigate the drop formation at the disk edge [94]. The results showed that a fine grooved disk is easier to yield droplets with a narrow size distribution, because a smooth liquid film forms on the disc edge and the liquid spreads uniformly. The size distributions for the disks with blocks and grooves and with pits are narrower than that for the flat disk, regardless of low or high rotation speeds, due to the existence of barriers on the surface to impede the spread of the liquid film. The grooved disk has a smaller droplet size than the pit disk. The effect of the edge structure of flat disks on the liquid film evolution and the droplet size was also assessed. The side wall adhesion phenomenon was observed due to the changed balance between the concurrent internal force maintaining the liquid film and external force tearing the liquid film over the rotating atomizer [95]. The different film thicknesses at the edge of the atomizers with different edge structures lead to a change in the critical volume flow rate, and hence the disintegration mode. The long-flat edge disk tends to have the smallest average droplet diameter, followed by the shot flat disk. Thinner disk type can produce slimmer and shorter filaments, resulting in a wider scatter of small droplets. While thicker disk edges produce larger filaments and a thicker stagnant layer, leading to more pronounced inward flight angles [96]. The scattering characteristics of the large terminal droplets show no significant discrepancy across different types, as shown in Figure 6. The existing correlations for the droplet size are summarized in Table 7.

The ligament mode can theoretically produce powder with a narrow particle size distribution because it transforms droplet generation from a random process into an orderly one as aforementioned. This allows the melt to be efficiently broken up into uniformly sized droplets, resulting in a narrower particle size distribution compared to direct droplet mode under ideal conditions, while simultaneously avoiding the chaotic state associated with sheet mode. It is the target operating regime for most industrial CA processes, but achieving and maintaining a pure, uniform ligament mode across the entire operating range is challenging. The process at industrial flow rates is likely in an unstable or mixed mode, oscillating between ligament mode and chaotic sheet mode, especially at high industrial flow rates [74]. The primary goal in industry is productivity. This requires high melt flow rates. As known from Equation (4), increasing the flow rate pushes the process away from direct drop mode and toward ligament mode and eventually sheet mode. Therefore, by design, industrial CA operations almost always run in a parameter space that is between ligament and sheet mode.

**Figure 7:**
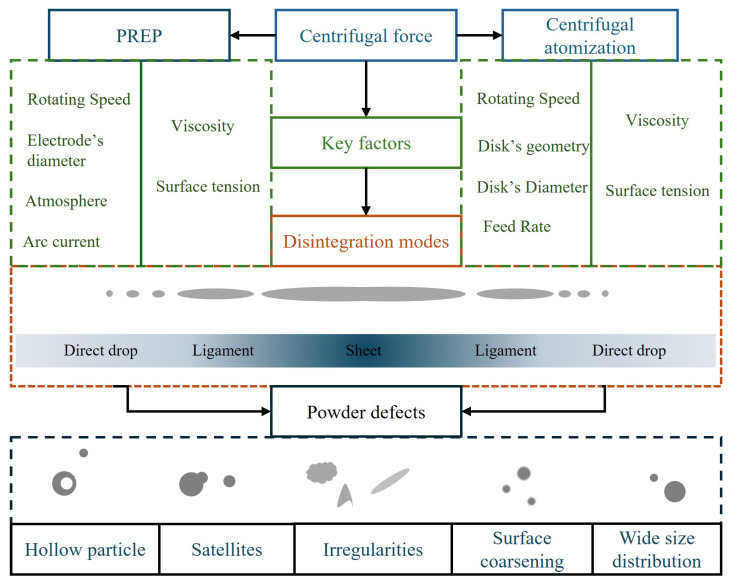
Schematic illustration of parameters affecting the disintegration mode and potential powder defect for PREP and CA.

Although both PREP and CA involve centrifugal force, there are fundamental differences in the underlying dominant physical mechanisms and the final products. For PREP, molten melt is ejected from the isolated edge of a rotating electrode. The formation and breakup of ligaments are dominated almost entirely by centrifugal force, surface tension, and inertial forces, with relatively minor influence from the surrounding gas drag force. For CA, in contrast, the melt is accelerated and ejected from the edge of a rotating disk. Ligament formation is not only influenced by centrifugal force but is more strongly governed by constraints such as the geometry of disk and surface wettability. This consequently leads to the differing sensitivities of PREP and CA to various dimensionless fluid parameters. Much research on the rotating electrode process indicates that mode transitions exhibit specific thresholds for melting rate and rotational speed. In Liu’s [81] study, it is mentioned that powder particle size is strongly correlated with the *We*, but is simultaneously strongly limited by the melting rate. Increasing *We* (increasing rotational speed) can refine powder, but this effect may be counteracted by a simultaneously increased melting rate of raw materials. Similarly, research by Tang et al. [17] showed that increasing rotational speed (increasing *We*) is the primary method for powder refinement, but the effectiveness curve is non-linear and exhibits a saturation trend, indicating the existence of an effective range for the *We*. Furthermore, in PREP, the melt is ejected from the electrode edge and solidifies into powder within an extremely short time, with its flight path in an inert gas. The lifetime of any ligament is extremely short, and viscous forces lack sufficient time to fully dissipate energy. Nie et al. [15] compared the PREP powder production process for various alloys. The study found that although the viscosities of different alloys (different *Oh*) differed, the particle size and morphology of the produced powders were very similar as long as their surface tension and density were comparable. This indicates that under the rapid solidification conditions of PREP, surface tension is a more critical factor than viscosity. In contrast, studies on ligament dynamics in CA clearly state that the final droplet diameter is directly related to the ligament diameter. *We* is a core design parameter for CA technology, with powder particle size being inversely proportional to the square root of the *We* [75]. The formation of ligaments is closely related to surface tension, density, and rotational speed. Moreover, high viscosity (high *Oh*) strongly suppresses the growth of instability waves and the necking process, resulting in thicker, more stable ligaments [105]. Figure 7 summarizes the parameters affecting the disintegration mode in PREP and CA processes and their potential powder defects.

## 4. Challenges and Insights for PREP and CA

PREP and CA technologies have been demonstrated to have mature capabilities for the production of powders that are suitable for PM and AM applications [106,107,108,109,110,111,112]. However, some particle defects and phenomena that can shorten the lifespan of the product or even the equipment may arise during the production process. Investigations into these defects and phenomena can assist in a better understanding of the principles and operational methods to improve the efficacy of the PREP and CA technologies.

### 4.1. Powder Defects

The quality of the powder, including the particle shape, relative density, particle size distribution, and so on, has significant effects on the final products and their applications. Compared to gas-atomized powders, PREP powders offer superior characteristics, including excellent sphericity, high purity, fewer satellites, virtually no hollow particles, and a significantly narrower particle size distribution [113,114]. However, PREP can still yield powders with defects due to the specific properties of certain raw materials or improper operating conditions.

**Figure 8:**
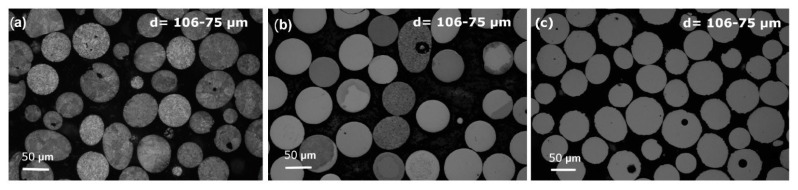
Pore formation in CA powder of (**a**) Fe_91.72_Si_5.32_B_2.96_; (**b**) Fe_87.37_Si_6.94_B_2.49_Cr_2.46_C_0.75_; (**c**) Fe_89.41_Si_2.02_B_1.13_P_5.89_C_1.55_ [115].

Pore formation in particles can occur in PREP and CA powders and two primary mechanisms are usually responsible for these types of defects (Figure 8). First, ejection of molten droplets from the electrode’s edge can result in entrapment of the high-pressure inert gas within the droplets in the atomization chamber. Second, the droplets and the surrounding inert gas interact with each other during flight before solidifying. This frictional interaction facilitates the entrainment of inert gas into the droplets, further contributing to pore formation. Surface tension plays a critical role in gas entrainment. It is the primary factor contributing to porosity variations among particles of different sizes. Gas is less likely to be captured in droplets exhibiting a higher surface tension, which is strongly influenced by temperature. Smaller droplets cool more rapidly than larger ones, leading to greater surface tension. Consequently, larger droplets exhibit a greater tendency for gas entrapment than finer droplets, ultimately yielding higher porosity levels in the coarser powder post-solidification [20]. The same trend was also observed for the manufacturing of TC4, 316L and CCM powder [18], increasing the rotation speed is an efficient way to eliminate pore formation due to smaller particle with higher surface tension.

Although a high degree of sphericity has always been one of the greatest advantages of PREP technology, non-spherical particles can still be present. Low particle sphericity is usually more prevalent in larger particles. Fan et al. [52] reported that the average particle sphericity for the Co_31.5_Cr_7_Fe_30_Ni_31.5_ powder with a particle size less than 50 μm is 96.26%, while the particle sphericity for particles over 106 μm is 76.14%. Since particle sphericity is sensitive to surface tension, the difference is mainly related to the degree of undercooling and the cooling rate of flying droplets during solidification. Small droplets with high undercooling are much easier to achieve a smooth surface structure after solidification. A similar result was reported in the Ti-48Al-3Nb-1.5Ta powder and irregularly shaped particles were present [59]. The cause of this was identified as premature solidification. The process involves centrifugal and shear forces detaching a liquid film from the surface. This semi-solid film then forms droplets that solidify mid-flight, preventing them from becoming spherical. Additional gas flowing plays an important role in powder manufacturing. During PREP production, increasing the yield of fine powder also enhances the sphericity of the powder. Utilizing a helium atmosphere instead of argon promotes a steeper incline on the electrode end face, enhancing droplet ejection and naturally yielding smaller powders with high sphericity. Moreover, an optimal coaxial flow of inert gas can be applied, as it creates a disturbance effect that crushes the molten film into finer particles. However, the flow rate must be carefully controlled to prevent the competing cooling effect from dominating and increasing powder size. These methods provide a more practical and effective approach than solely relying on increasing rotational speed [29,30,31].

**Figure 9:**
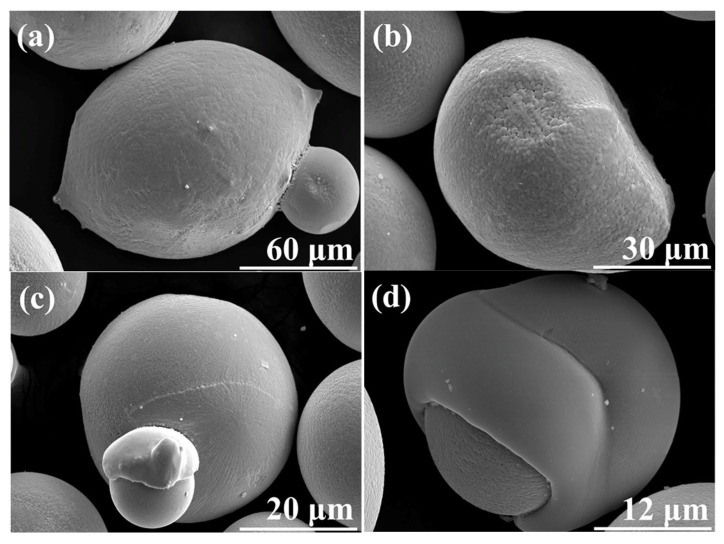
Particle defect (**a**) satellite powder; (**b**) nonspherical particle; (**c**) collision particle; and (**d**) irregular particle in PREP powder [12].

Satellite particles are another powder defect that decreases the particle sphericity and hence reduces the powder flowability, as shown in Figure 9. Satellite particles form when flying droplets at different solidification stages collide. Specifically, a hotter, softer (slower-solidifying) droplet collides with a cooler, harder (faster-solidifying) one, embedding the latter into the former’s surface. The bonding between the parent particle and its satellite can have a clear or blurred interface or form a neck, depending on the solidification state and the size of the two particles upon collision [15]. PREP and CA exhibit a notably lower frequency of satellite powder formation compared with other atomization techniques. This is attributed to two reasons: the droplet breakup mechanism induced by centrifugal force, which creates relatively uniform flight trajectories, and the sufficiently long flight path that ensures most particles solidify completely before collision. Research on satellite formation in PREP and CA consistently identifies one primary condition: satellite particles form when collisions occur between droplets, with at least one remaining in a molten or semi-molten state [25]. This phenomenon can be effectively mitigated by increasing the rotational speed [17].

The particles of the powder manufactured by CA are more prone to being irregular than PREP powders. The formation of flakes is attributable to the impact of inadequately cooled droplets on the quench wall. Herein, the residual heat renders the particle incapable of retaining its sphericity upon impact, leading to deformation under stress. Consequently, providing a longer flight path to facilitate sufficient cooling prior to impact is an effective measure for mitigating flake formation. Another study [53] showed that particles with sizes more than 125 μm have irregular shapes. Observations using a high-speed camera showed that the origin of the irregular particles was from the edge of the atomizer, where a solid layer formed due to the uneven temperature distribution over the surface. Generation of irregular particles is not due to ligament disintegration [116]. The probability of irregular particle formation increases significantly with insufficient rotational speed, while flakes only appear with rotation speeds over 10,000 rpm. Adjusting the shape and edge structure of the atomization disk can effectively improve powder quality, as mentioned in Section 3. Compared to conical and biconical disks, the spherical disk produces atomized powder with relatively smaller particle size, narrower size distribution, and higher yield [117]. Angers et al. [99,118,119] found that using a concave disk improves metal contact and reduces cooling, minimizing solidification. Furthermore, a multi-layer graded coating protects the disk from thermal shock and corrosion while improving wetting. This leads to more consistent droplet formation and higher powder sphericity.

Surface peeling was found in the residual electrode surface in PREP, especially for alloys with high thermal conductivity, causing an increase of the coarseness of the powder particles [15,17,110]. Surface peeling is likely associated with the shear forces induced by the cooling atmosphere on the liquid film. The outer region of the film, which experiences the highest centrifugal and shear forces, exhibits more severe spalling. The morphology of the semi-solidified liquid film fragments may already be determined prior to their detachment from the rest of the film. As a result, they form irregularly shaped particles instead of assuming a perfectly spherical shape under surface tension.

### 4.2. Limitations for PREP and CA

PREP can produce reactive powder with high purity, because it is a ceramic-free process, eliminating the risk of ceramic contamination from the crucible as used in the other atomization methods. A primary drawback of PREP is its long production cycle. This is because the feedstock must first be cast as a large ingot and then machined into a precisely dimensioned rod. This additional machining, necessary for high-speed rotation, further increases the overall production cost. The size limitation of the anode bar makes it difficult to yield fine powder. The particle sizes of Ti6Al4V powders manufactured by PREP are usually between 100 and 300 μm, with a median diameter of about 175 μm, which is way too big for many applications [120]. Reduction of particle size is possible by increasing the diameter of the feedstock bar and optimizing the operating parameters. For example, the median particle size of Ti6Al4V was decreased from 312 to 168 μm by increasing the rotating speed from 6000 rev/min to 12,000 rev/min [121]. Increasing the melting rate of the material increased the chance of producing more ligament disintegration, leading to finer Ni-based alloy particles [81]. However, one should be aware that larger electrode diameters and higher rotational speeds impose stricter dimensional accuracy requirements on the electrode to reduce unbalanced forces. It is not always feasible and, even if possible, can incur a high cost.

**Figure 10:**
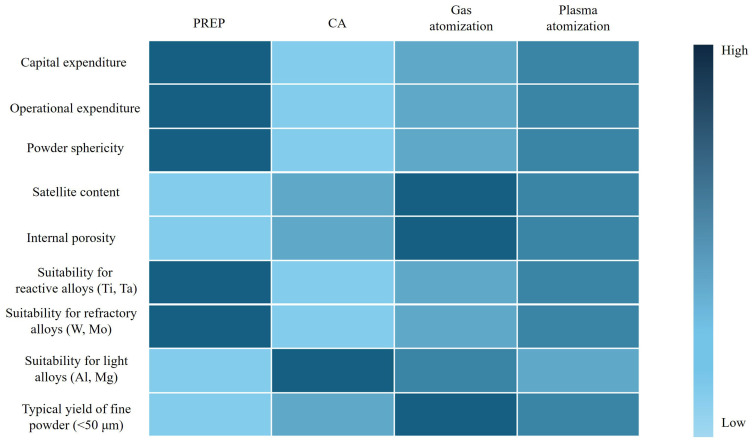
Comparisons among PREP, CA, gas atomization and plasma atomization.

CA is widely employed due to its operational simplicity, low cost and wide applicability, compared to the other atomization processes. However, it has one major problem, namely the formation of a skull on the surface of the atomizer. This phenomenon refers to the formation of a solidified layer (skull) of molten metal on the atomizer due to rapid cooling. This occurs when the molten metal comes into contact with the cooler surface of the atomizer before being ejected into droplet. The thickness of the skull is not constant but becomes progressively thicker from the center of the atomizer toward the edge. The inner area close to the center is thinner due to two effects: (1) the impingement of the liquid metal stream provides a constant input of heat; (2) the shrinkage and warping of the solidified skull layer creates a gap with the atomizer, reducing heat dissipation to the atomizer [81]. The sharp increase in skull thickness can be attributed to the heat conduction to the motor shaft and convective cooling by the surrounding atmosphere [122]. A computational fluid dynamics model has been developed to simulate the growth of the skull until an equilibrium state is reached [123]. Unsurprisingly, the solidification of the liquid is delayed with increasing liquid metal flow rate, initial disk and liquid temperatures. Increasing wettability between liquid and atomizer can reduce the formation of irregular particles but promote the occurrence of skull simultaneously. Gonsrang et al. [124] found it a good way to remove the skull on the atomizer by preheating it prior to atomization. A problem with skull formation is that the skull can detach from the atomizer during operation due to centrifugal force. Skull detachment reduces the service life of the equipment and, in severe cases, can directly damage the machine, and needs to be avoided. For both PREP and CA, when compared to gas atomization, they possess irreplaceable advantages as well as drawbacks that need to be overcome. Figure 10 illustrates the comparisons among the four technologies (PREP, CA, gas atomization and plasma atomization) in terms of cost, fine powder yield, powder quality, and suitability for different alloys.

## 5. Summary and Perspective

### 5.1. Scalability Challenges for Industrial Adoption

PREP and CA are proven to have the capability to produce high-purity, spherical powders via centrifugal force. However, they also face significant challenges for mass industrial adoption. The principal challenge lies in their relatively low yield of fine powders (typically below 50 µm), which are crucial for advanced manufacturing processes like laser powder bed fusion and metal injection molding. Scaling up throughput necessitates larger electrodes or higher rotational speeds, which in turn exponentially increases equipment complexity, maintenance costs, and energy consumption. PREP is indispensable for oxygen-sensitive superalloys [125], although its low yield of fine powder and high capital expenditure limit its scalability compared to gas atomization. For CA, although it is appropriate for metals with low melting point like Al, Sn, and Pb, it faces significant challenges in maintaining consistent powder shape and preventing skull formation at high volumes.

### 5.2. Potential Integration with Advanced AM Feedstock Requirements

The integration of PREP and CA with AM demands a focus on advanced quality. PREP is exceptionally suited for critical AM applications in aerospace and biomedical sectors, where its rarely hollow particle and satellite-free powder directly led to superior mechanical performance and part density in AM-fabricated components [126,127]. To better align with AM feedstock needs, process innovations are critical. These include replacing conventional electron beams with high-power laser or plasma arc sources for more efficient melting [116], utilizing magnetic field assistance to enhance arc penetration [128], employing techniques like the pulsated orifice ejection method to improve melt wettability on the disk and enhance sphericity [129], and employing metal-cored arc welding with a rotating electrode process, which allows the electric arc to reach peripheral regions of the groove, preventing lack of fusion on the sidewall [130].

### 5.3. Prospects for Metallic Glasses and Composites

The future of PREP and CA is intrinsically linked to their ability to process advanced material systems. Their rapid solidification characteristics are a significant advantage for producing various materials. An Al_86_Ni_8_Y_4.5_La_1.5_ glass-forming alloy powder with particle sizes < 125 μm, having an amorphous fraction above 70%, was fabricated successfully using a He atmosphere [131]. The ratio of amorphous to crystalline structures in the powder is mainly dependent on the cooling rate, which can be modulated by adjusting processing parameters, altering gas atmosphere, and controlling powder particle size [115]. Moreover, the feasibility of CA for fabricating composite materials has also been validated experimentally. An Al/SiC composite powder with a narrow grain size distribution was manufactured, although the degree of particle sphericity is poor [132]. The impingement of SiC particles seems to enhance the breakup of the large ligaments into small ligaments. The microstructure can be altered by adjusting the operating parameters, since a high cooling rate can lead to finer and more homogeneous dendrites instead of equiaxed structures [60]. Overall, this tunability opens up new possibilities for developing novel materials.

### 5.4. Environmental and Energy Efficiency Considerations

Addressing the high energy footprint of PREP and CA is paramount for their sustainable adoption. Current research focuses on minimizing energy consumption by transitioning from electron beams to more efficient high-power laser or plasma arc heat sources [116,133]. Furthermore, the industry is actively exploring recycling and repurposing of process byproducts, such as oversize powder fractions, to improve overall material yield and reduce waste. Some of the inherent difficulties, such as low fine-powder yield, underused byproducts, and large equipment with high maintenance costs, are gradually being addressed by coordinated efforts in both the industrial and academic domains. Nevertheless, sustained interdisciplinary collaboration and innovation are still necessary to enhance their energy efficiency and to expand their feasibility for the production of powders from advanced materials.

## Figures and Tables

**Table 2:** The physical characteristics of varying PREP-ed powder.

Type	Particle Size (μm)	Flowability (s/50 g)	Apparent Density (g/cm^3^)	Tap Density (g/cm^3^)	Ref.
Ti6Al4V	60–120	29.6	2.59	-	[20]
W	45–150	6	11.35	12.5	[21]
W-0.3%La_2_O_3_	45–150	6.4	10.91	11.62	
W-1%La_2_O_3_	45–150	6.33	10.85	11.24	
Ni-11Mo-8Al-3Ta-2Cr-1Re	<50	13.7	4.83	5.15	[13]
	50–150	12.2	4.86	5.24	
S800 Ag	23.08–51.04	-	5.92	6.2	[22]
Ti6Al4V	<45	23.17	-	-	[23]
	100–150	26.61			
	178–250	28.48			
Ti-28at.%Ta	30–260	15.5	-	-	[24]
IN718	26.5–39.9	12.4	4.84	-	[25]
EP648	55–110	17.08	4.8	-	[26]
	110–170	18.36	4.84		
IC21	<53	14.47	4.72	5.24	[19]
	53–106	14.92	4.76	5.21	
K390	<150	13.51	4.89	6.13	[27]
T15 high speed steel	<250	11.7	5.17	5.5	[28]

**Table 3:** Summary of particle sizes and corresponding processing parameters for various PREP powders.

Type	Average Particle Size (μm)	Rotational Speed (rpm)	Diameter of the Electrode (mm)	Arc Current (A)	Gas Type	Gas Flow Rate (L/min)	Ref.
Ti64	~550	7000	15	80	Ar	-	[29]
	~450	9000					
	~400	11,700					
	~500	7000	20				
	~410	9000					
	~325	11,700					
	~400	7000	25				
	~350	9000					
	~250	11,700					
Ti64	~580	7000	15	50	Ar	-	[30]
	~600			70			
	~650			90			
	~378	-	15	80	Ar	0	
	~370					50	
	~350					100	
	~380					150	
	~340				He	0	
	~325					50	
	~348					100	
	~350					150	
Ti64	~640	7000	15	80	Ar	-	[31]
	~480	9000					
	~380	11,700					
	~500	7000	20				
	~430	9000					
	~350	11,700					
	~450	7000	25				
	~330	9000					
	~280	11,700					
	371.3	11,700	15			0	
	384.4					70	
	421.7					160	
SUS316	~320	7000	20			-	
	~260	9000					
	~200	11,700					
	~280	7000	20	50			
	~350			70			
	~365			90			
Ti6Al4V	240 (D50)	8000	75	-	Ar	-	[17]
	226 (D50)	10,000					
	185 (D50)	12,000					
	156 (D50)	14,000					
Ti-45Al-7Nb-0.3W	46–150 (full range)	15,000–16,000	75	1100	Ar	-	[33]
Ti-48Al-2Nb-2Cr	25–150 (full range)	32,000–42,000	30	650–700	-	-	[16]
Ti-28 at.%Ta	30–260 (full range)	12,000	50	1100	-	-	[24]
IN600	70–130 (full range)	-	-	-	Ar	-	[34]
S800 Ag	15–60 (full range)	25,000–37,000	30	500–700	Ar	-	[22]
Co31.5Cr7Fe30Ni31.5	20–106 (full range)	23,000–32,000	29	760		-	[35]
316H	51.3 (D50)	10,000–35,000	50	1200–1550	Ar	-	[36]

**Table 4:** Summary of particle sizes and corresponding processing parameters for various CA powders.

Type	Average Powder Size (μm)	Rotating Atomizer	Melt Feed Rate (kg/h)	Diameter of Atomizer (mm)	Rotating Speed (rpm)	Melt Temperature (°C)	Ref.
Al	107.9 (D50)	12° tapered	2.94 mL/s	59	18,000	-	[40]
	99.1 (D50)				24,000		
	90.2 (D50)				33,000		
	80.2 (D50)				39,000		
	266.8 (D50)	R90 curved	5.33 mL/s	59	6000	-	
	179 (D50)				12,000		
	147.7 (D50)				18,000		
	116 (D50)				24,000		
	85.5 (D50)	R80 curved	2.94 mL/s	59	24,000	-	
	99.7 (D50)	Flat					
Cu	88.3 (D50)	Disk	75	40	15,000	1200	[41]
	72.4 (D50)				25,000		
	68.1 (D50)				35,000		
SAC305	~90/~95	Flat disk	32	50	10,000	318	[42]
	~65/~67				15,000		
	~60/~70				20,000		
	~45/~50				25,000		
	~44/~49				30,000		
	~50/~56		17.7		20,000		
	~60/~70		31.3				
	~70/~80		64.7				
	~130/145		133.6				
	~110/~130	Flat disk	63.3	30			
	~105/~115			40			
	~70/~80			50			
	~108/~112	Cup	63.3	30	20,000		
	~85/~90			40			
	~70/~75			50			
Al-4%Cu	113 (D50 with Ar)	Disk	-	45	40,000	776.85	[43]
	107 (D50 with Ar)					576.85	
	119 (D50 with He)					776.85	
	104 (D50 with He)					576.85	
Nd-Fe-B alloy	320 (thickness)	Wheel	-	-	210	~1452	[44]
	120 (thickness)				280		
Ca	126.1/123.8 (D50) 168.2/181.8 (D50)	Cup	-	51	20,000	-	[45]
Semisteel	550	Cup	1.3 × 10^−5^ m^3^/s	110	600	1450	[46]
	~525				800		
	490				1000		
Gd	216 (D50)	Disk	-	41	8000	1433	[47]
Zn	~275 (D50)	Disk	50.47	40	10,000	550	[48]
	~250 (D50)				15,000		
	~200 (D50)				20,000		
	~150 (D50)				25,000		
	~125 (D50)				30,000		
	~160 (D50)			30			
	~110 (D50)			50			
	~95 (D50)			60			
	~100 (D50)		81.66				
	~105 (D50)		121.38				

**Table 5:** Summary of particle sizes and corresponding processing parameters for various powders.

Type	Particle Size (μm)	SDAS (μm)	Cooling Rate (K/s)	Atmosphere	Ref.
Al_0.5_CoCrFeNi	25–50	0.14–0.37	10^5^–10^6^	Argon	[12]
	50–75	0.37–0.63			
	75–100	0.63–0.89			
	>100	0.89			
Al-4%Cu	~165	~2	10^4^–10^5^	Helium	[43]
		~2			
		~4.2	10^3^–10^4^	Argon	
		~4.5			
Ti-28Ta	~50	~1.05	-	-	[24]
	~60	~1.1			
	~70	~1.1			
	~85	~1.4			
	~95	~1.4			
	~100	~1.5			
	~110	~1.6			
Ni-11Mo-8Al-3Ta-2Cr-1Re	<150	2–4	10^5^–10^7^	Ar/He mixture	[13]
Ti-60Ta	~50	~1	10^4^–10^6^	Argon	[58]
	~60	~1.1			
	~70	~1.2			
	~85	~1.4			
	~100	~1.4			
	~105	~1.5			
	~110	~1.6			
Ti-48Al-3Nb-1.5Ta	~80	~2	~17,000	Ar/He mixture	[59]
	~110	~2.5	~10,000		
	~135	~4.25	~8000		
	~170	~4.75	~6000		
AlCu4Mg1-SiC	~47	0.85	10^−3^–10^9^	Argon	[60]
	~250	4.16			
	~450	9.97			

**Table 6:** Criteria of transition between different disintegration modes and practical implications for PREP and CA.

Atomizer	Disintegration Mode	Critical Conditions	Practical Implications	Ref.
CA (disk)	Direct drop-Ligament	$Q<1.52\left(\frac{\rho \sigma D}{\mu^{2}}\right)\left(\frac{\mu D}{\rho }\right)/{\left(\frac{\omega \rho D^{2}}{\mu }\right)}^{0.95}$	Large, spherical droplets. Coarse powder production or low-throughput applications.	[82]
		$Q^{*}=0.308{We}^{-0.994}{Re}^{0.201}$		[72]
	Ligament	$Q>0.46{\left(\frac{\rho \sigma D}{\mu^{2}}\right)}^{0.9}\left(\frac{\mu D}{\rho }\right)/{\left(\frac{\omega \rho D^{2}}{\mu }\right)}^{0.63}$	Spherical to slightly oval particles. Balance between speed and feed rate to stabilize ligaments.	[82]
	Ligament-Full ligament	$Q^{*}=0.126{We}^{-0.779}{Re}^{0.118}$		[72]
	Ligament-Sheet	$Q>19.8{\left(\frac{\rho \sigma D}{\mu^{2}}\right)}^{0.9}\left(\frac{\mu D}{\rho }\right)/{\left(\frac{\omega \rho D^{2}}{\mu }\right)}^{0.84}$	A mix of spherical and irregular (satellite) particles. Increased satellite formation from sheet breakup. Optimize *We* to minimize unstable sheet formation.	[82]
		$Q^{*}=0.257{We}^{-0.75}{Re}^{0.133}$		[72]
CA (cup)	Direct drop-Ligament	$Q^{*}=6.5{We}^{-1.161}{St}^{-0.0705}$	Suitable for a wide range of metal powders. Offers a good balance between yield and particle shape control.	[77]
		${\left[\omega \sqrt{\frac{\rho D^{3}}{\sigma }}\right]}^{0.25}Q\sqrt{\frac{\rho }{\sigma D^{3}}}{\left(\frac{\mu }{\sqrt{\rho \sigma D}}\right)}^{1/6}=2.88\times 10^{-3}$		[75]
	Ligament-Sheet	$Q^{*}=5.13{We}^{-0.789}{St}^{0.036}$	Enables high production rates. High likelihood of satellite and fine powder formation. Use this mode intentionally for applications requiring fines but expect a broader size distribution.	[77]
		$\left(\frac{\rho_{L}\omega^{2}D^{2}}{\sigma }\right){\left(\frac{Q}{\omega D^{3}}\right)}^{\frac{4}{3}}{\left(\frac{v_{s}d_{L}}{Q}\right)}^{0.19}>0.363$		[83]
PREP	Direct drop	Hi<0.07	Highly spherical, satellite-free powders. Premium applications require superior flowability.	[29,31,78,79,80,81]
	Ligament	0.07≤Hi≤1.33	Ligaments breakup into droplets via Rayleigh–Plateau instability, which can lead to larger powder size and a broader particle size distribution	
	Sheet	Hi>1.33	Large and irregularly shaped droplets formed. It should be avoided for high-quality powder production.	

**Table 7:** Correlations for droplet size in PREP and CA.

Atomizer	Correlation	Disintegration Mode	Remarks	Ref.
PREP	$d=\frac{1}{\omega }\sqrt{\frac{12\gamma }{\rho D}}$	Direct drop-ligament		[29,81]
PREP	$d=2.0\frac{1}{\omega }\sqrt{\frac{\gamma }{\rho D}}$	Ligament		[78,82]
Disk	$\frac{d}{R}=0.468{We}^{-0.056}{St}^{0.155}Q_{R}^{0.465}$	All modes	$Q_{R}=\frac{\rho Q^{2}}{\gamma R^{3}}$	[97]
Slotted	$\frac{d}{R}=0.900{We}^{-0.195}{St}^{0.214}Q_{R}^{0.223}$		$We:1.40\times 10^{3}\sim 1.04\times 10^{5}$ $St:1.38\times 10^{-3}\sim 1.39\times 10^{-2}$	
Arc-edge	$\frac{d}{R}=0.600{We}^{-0.147}{St}^{0.158}Q_{R}^{0.369}$		-	
Bulged block	$\frac{d}{R}=0.429{We}^{-0.026}{St}^{0.184}Q_{R}^{0.526}$		$We:2.61\times 10^{-3}\sim 9.22\times 10^{-2}$	
Disk/Cup	$\frac{d_{32}}{R}=0.84{\left(\frac{H}{R}\right)}^{0.26}{\left(We\right)}^{-0.28}{\left(Re\right)}^{0.27}{\left(Oh\right)}^{0.38}$	All modes	*H* is the depth of the atomizer.	[98]
Disk	$d=4.27\times 10^{6}\cdot \frac{1}{\omega^{0.95}}\cdot \frac{1}{D^{0.61}}\cdot {\left(\frac{\gamma }{\rho }\right)}^{0.42}\cdot Q^{0.12}$	All modes	-	[99]
Cup	$d=d_{drop}{\left(\frac{\rho_{liquid}}{\rho_{solid}}\right)}^{3/2}$	All modes	$d_{liquid}={\left[\frac{3}{2}2^{\frac{1}{s}}\pi d_{L}^{3}\left(1+3Oh\right)\right]}^{3/2}$	[73]
Cup	$\frac{d}{R}=2.753{We}^{-1/2}$	Direct drop mode	-	[77]
Disk	$d={\left(\frac{3\pi }{\sqrt{2}}\right)}^{1/3}d_{L}{\left(1+\frac{3}{\sqrt{\rho \gamma d_{L}}}\right)}^{1/6}$	All modes	We:2000~28,000	[100]
Disk	$\frac{d}{R}=2.582{We}^{-0.321}{Oh}^{0.251}Q_{R}^{1/7}$	All modes	$We:0.815\times 10^{3}\sim 2.47\times 10^{5}$ $St:6.76\times 10^{-6}\sim 1.43\times 10^{-4}$ $Q_{R}=0.005\sim 0.056$	[101]
Disk	$\frac{d_{32}}{R}=2.901{Re}^{0.493}{We}^{-0.4926}{Oh}^{0.3693}$	Sheet mode	Re:43.4~1540.2 We:8979.6~181,373.2 Oh:0.0004996~0.0065497	[87]
Disk	$d=d_{droplet}\sqrt[{3}]{\frac{\rho_{liquid}}{\rho_{solid}}}$	Sheet mode	λ_opt_ is the optimal wavelength of the wave on the ligament $d_{droplet}=\sqrt[{3}]{\frac{3}{2}\lambda_{opt}d_{l}^{2}}$	[102]
PREP	$D_{ligament}=0.5774D_{direct\;drop}$	Direct drop-ligament		[81]
Cup	$\frac{d}{R}=1.628{We}^{-0.312}{Re}^{0.240}{Oh}^{0.4043}$	All modes	We:24,604~338,143 Re:3.1~33.8 Oh:0.045~0.168	[103]
Cup	$\frac{d}{R}=3.3459{We}^{0.2767}{Re}^{0.3938}{Oh}^{0.7479}{\left(\frac{Q}{\rho \omega R^{3}}\right)}^{0.5301}{\left(\frac{R_{L}}{R}\right)}^{1.7714}$	All modes	Re:207.95748~584.0870 We:1592.1762~2081.87 Oh:0.078118~0.204692 Q:0.011318~0.022635 $\frac{R_{L}}{R}:0.2\sim 0.6$	[104]

## Data Availability

No new data were created or analyzed in this study. Data sharing is not applicable to this article.
